# Potential Benefit of the Charge-Stabilized Nanostructure Saline RNS60 for Myelin Maintenance and Repair

**DOI:** 10.1038/srep30020

**Published:** 2016-07-25

**Authors:** Vijayaraghava T. S. Rao, Damla Khan, Russell G. Jones, Diane S. Nakamura, Timothy E. Kennedy, Qiao-Ling Cui, Malena B. Rone, Luke M. Healy, Richard Watson, Supurna Ghosh, Jack P. Antel

**Affiliations:** 1 Montreal Neurological Institute, McGill University, Montreal, Quebec, H3A 2B4, Canada; 2 Department of Physiology, McGill University, Montréal, Quebec, Canada; 3 Revalesio Corporation, Tacoma, Washington, USA

## Abstract

Myelin injury in multiple sclerosis (MS) has been attributed both to “outside-in” primary immune mediated and “inside-out” metabolic stress of oligodendrocyte (OL) related mechanisms. Subsequent remyelination is dependent on recruitment and differentiation of oligodendrocyte progenitor cells (OPCs). RNS60 is a physically-modified saline containing charge-stabilized nanobubbles generated by subjecting normal saline to Taylor-Couette-Poiseuille (TCP) flow under elevated oxygen pressure. Administration of RNS60 has been shown to reduce the severity of EAE by dampening the immune response and myelin loss. Additionally, RNS60 has been demonstrated to enhance mitochondrial ATP synthesis in neurons. Here, we used post-natal rat derived OLs and OPCs to assess the impact of RNS60 on the response of OLs to metabolic stress *in vitro* (glucose-nutrient deprivation, referred to as ‘NG’) and on OPC differentiation capacity. Under the NG condition, our findings indicate that RNS60 decreases caspases 3/7 activation. Respirometric analyses revealed that RNS60 increased spare glycolytic capacity (SGC) under normal culture conditions. However, RNS60 enhanced OL spare respiratory capacity (SRC) when a metabolic stress was present. Furthermore, we show that RNS60 promotes OPC differentiation under physiological conditions. Our findings provide evidence for the potential therapeutic efficacy of RNS60 through the promotion of OL survival and OPC differentiation.

Multiple sclerosis is characterized by multi-focal demyelinating lesions with variable extent of subsequent remyelination. There is pathologic heterogeneity in acute lesions amongst different cases, with subtypes considered to reflect “outside-in” and “inside-out” mechanisms of tissue injury. The former is ascribed to recruitment of myelin directed auto-reactive T cells and antibodies to the CNS, as occurs in the animal model of experimental autoimmune (allergic) encephalomyelitis (EAE)^1^. The “inside-out” mechanism implicates local metabolic derangements, which include oligodendrocyte (OL) process retraction and cell death^2^. Histological support for the latter form is provided by observations of expression of ischemia associated molecules in such lesions^3^. Over time there is continued oligodendrocyte loss in MS lesions, a process compatible with continuous stress on these cells regardless of the initiating event. Neuro-protective strategies in MS, and animal models of MS, include the use of agents such as anti-oxidants that could act upon both oligodendrocytes and neurons^4^. Remyelination in the CNS is ascribed to recruitment and differentiation of oligodendrocyte progenitor cells (OPCs) rather than to previously myelinating oligodendrocytes (OLs)^5^. In the context of MS, OPCs in the lesion would be exposed to similar metabolic insults as the myelinating OLs. OPC numbers are increased at the margins of acute MS lesions but are decreased in chronic lesions^6^. The above observations present the challenge of identifying agents that would enhance resistance of both myelinating OLs and OPCs to metabolic stress and/or promote repair resulting from such insults.

RNS60 is a physically-modified saline containing charge-stabilized nanobubbles generated by subjecting normal saline to Taylor-Couette-Poiseuille (TCP) flow under elevated oxygen pressure. RNS60 administration has been found to reduce the severity of EAE. These effects were attributed to reduced pro-inflammatory responses in microglia consequent to suppression of nuclear factor-κB activation^7^, augmented T regulatory cell activity due to decreased Nitric oxide (NO) production, and suppressed differentiation of Th17 and Th1 cells^8^. Consequently, immune cell infiltration and loss of myelin were also decreased. RNS60 may also have direct effects on cells in the CNS. For example, Choi *et al*. demonstrated that RNS60 enhances mitochondrial ATP synthesis in squid neurons^9^.

We have previously documented that human OLs under conditions of metabolic stress *in vitro* initially show process retraction that is then followed by cell death^10^. This early stage is characterized by a significant reduction in overall energy utilization, particularly in glycolytic ATP production^11^. In the current study, we assessed the capacity of RNS60 to protect OLs derived from post natal rat OPCs from metabolic stress induced injury *in vitro*, using activation of caspase 3/7 as an injury read-out. To simulate metabolic stress OLs were subjected to glucose and nutrient deprivation. Since RNS60 contains oxygenated nanobubbles, we speculated that the survival effect may reflect its efficacy on OL respiration. We therefore used a Seahorse XF analyzer to assess the effect of RNS60 on the energy utilization properties of OLs under basal (control) and glucose-nutrient deprivation conditions. Further, we assessed whether RNS60 could promote the differentiation of OPCs into OLs.

## Materials and Methods

### Rodent OLs Cultures

Sprague-Dawley rats were used for this study. All experimental procedures using animals were performed in accordance with appropriate guidelines. All procedures were performed in accordance with the Canadian council on animal care guidelines for the use of animals in research and were approved by the Montréal Neurological Institute’s Animal Care Committee. OPCs derived from post-natal rats were collected by shake-off and differential adhesion after 8–14 days in mixed glial cell cultures^12^. The cells were grown in proliferation medium [Dulbecco modified Eagle medium/F12 supplemented with N1, 0.01% bovine serum albumin, 1% penicillin-streptomycin, B27 supplement, platelet derived growth factor (PDGF-AA), fibroblast derived growth factor (bFGF) and T3] for the first five days after plating. The cells were then cultured in differentiation medium [Dulbecco modified Eagle medium/F12 supplemented with N1, 0.01% bovine serum albumin, 1% penicillin-streptomycin, B27 supplement, 1% horse serum and T3; without PDGF-AA and bFGF]^13^.

#### Metabolic stress induced caspase activation

Differentiated OLs were cultured in Dulbecco’s Modified Eagle’s Medium (DMEM) [Sigma] without phenol red supplemented with GlutaMax (Life Technologies catalog no. 35050) and high glucose (at 4500 mg/L) for control condition. This basal/control condition herein will be referred to as ‘G’. OLs subjected to the metabolic stress condition were cultured in DMEM (Sigma) without phenol red supplemented only with GlutaMax. This stress condition herein will be referred to as ‘NG’. The NG condition was also performed in presence 10% v/v RNS60 that herein will be referred to as ‘NGRNS60’. Activation of Caspases 3/7 was assayed in these cultures after 35 h using CellEvent Caspase-3/7 Green reagent (ThermoFisher). DMEM devoid of phenol red is a necessary condition for the use of CellEvent Caspase-3/7 Green reagent. The cells were live stained for O4 along with Cell Event Caspase-3/7 Green reagent, fixed, followed by incubation with goat anti-mouse IgM Cy3 labeled secondary antibody (for O4) and DAPI. Immunostained cultures were photographed and caspase3/7- O4 double positive cells were counted.

### Respirometry (Extracellular Flux Analysis)

Rat OPCs were seeded at a density of 5,000–10,000 cells per well in Seahorse XF96 cell culture plates and allowed to differentiate into OLs as described above. 24 hours before respirometry, OLs were cultured in control/basal and metabolic stress conditions in DMEM as described above. Two hours before running the assay, control cultures were transferred to Seahorse XF assay medium with GlutaMax and high glucose (at 4500 mg/L) but lacking serum and sodium bicarbonate. This control condition herein referred to as ‘XF’. At the same time, OLs cultured under metabolic stress condition were transferred in to Seahorse XF assay medium with GlutaMax but lacking glucose, serum and sodium bicarbonate. This condition herein referred to as ‘XFNG’ condition. Both of these conditions were also carried out in presence of 10%v/v RNS60 herein will be referred to as ‘XFRNS60’ and ‘XFNGRNS60’ respectively. RNS60 was added once at the initiation of culturing in DMEM medium and a second time 24 hours later when cultures were transferred into XF assay medium. This transfer of cells into XF assay medium was done just a few hours before the XF assays were run. Oxygen consumption rate (OCR) and extracellular acidification rate (ECAR) were measured in XF-96 Extracellular Flux Analyzer, Seahorse Bioscience. The assay was performed wherein 4 basal cycles were included consisting of a 3 minutes mixing followed by 3 minutes of measurement. After completion, oligomycin (0.5 μM injection volume) was added for 3 cycles. Carbonyl cyanide-4-(trifluoromethoxy) phenylhydrazone (FCCP; 0.5 μM) was then added for an additional 3 cycles followed by rotenone (1 μM) plus antimycin A (2 μM) for another 3 cycles^14^,^15^. Extracellular acidification rates were calculated by the addition of 2-deoxy-glucose (2DG; 1M). All reagents were purchased from Sigma (St. Louis, MO). The OCR and ECAR data was normalized using protein concentration that was assayed with Pierce BSA kit (Thermofisher).

#### Calculation of spare respiratory capacity (SRC) and spare glycolytic capacity (SGC)

SRC was calculated from the OCR respirogram as the difference in OCR between the basal rate and the rate achieved following the addition of FCCP; this value was normalized to the basal rate. SGC was calculated from the ECAR respirogram as the difference in ECAR between the basal rate and the rate achieved following the addition of oligomycin; this value was normalized to the basal rate.

#### Efficacy of RNS60 to promote survival, proliferation, and differentiation of oligodendrocyte precursor cells (OPCs)

OPCs derived from post-natal rats were collected by shake-off and differential adhesion after 8–14 days in mixed glial cell cultures (McCarthy and de Vellis, 1980). The cells were then cultured in OL defined medium (DMEM, 5 μg/mL insulin, 100 μg/mL transferrin, 30 nM sodium selenite, 30 nM tri-iodothyronine, 2 mM glutamax)^16^. The cultures were assayed for the extent of differentiation by immunostaining for O4 and galactocerebroside (GalC) after 24 h in culture and for O4 and myelin basic protein (MBP) after 5 days in culture. For the cultures that were analysed after 24 h, 10% RNS60 was applied only at the time of plating. For the 5 days experiment, the reagents were applied at the initial plating of the cells and again 24 h later. 7,000 cells per well and 20,000 cells per well were the plating densities employed in 24 well dishes.

#### Immunocytochemistry

Anti-O4 and anti-GalC antibodies were prepared from cell lines. Anti-myelin basic protein (MBP) antibody was from Stemberger, Lutherville, MD, USA. Appropriate secondary antibodies were employed as previously published (20720504).

### Statistical analysis

**Figure 1:**
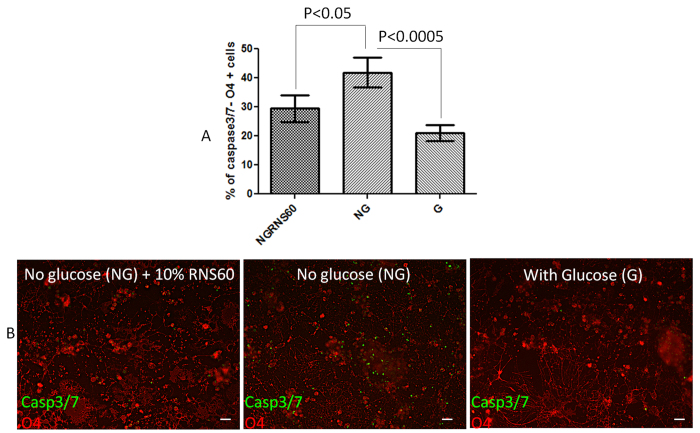
RNS60 promotes OL survival when challenged with glucose deprivation. The average percentage of total cells activating caspase3/7 was lower in the NGRNS60 condition compared to the glucose deprivation condition (NG). (**A**) Graph depicting the average percentage of total cells that were double positive for caspase3/7 and O4. NG RNS60 = No-glucose with 10% v/v RNS60, NG = No-glucose, G = With glucose. (**B**) Micrographs depicting OLs stained for activated caspase3/7 (green) and O4 (red). [Scale bar ~100 μm].

**Figure 2:**
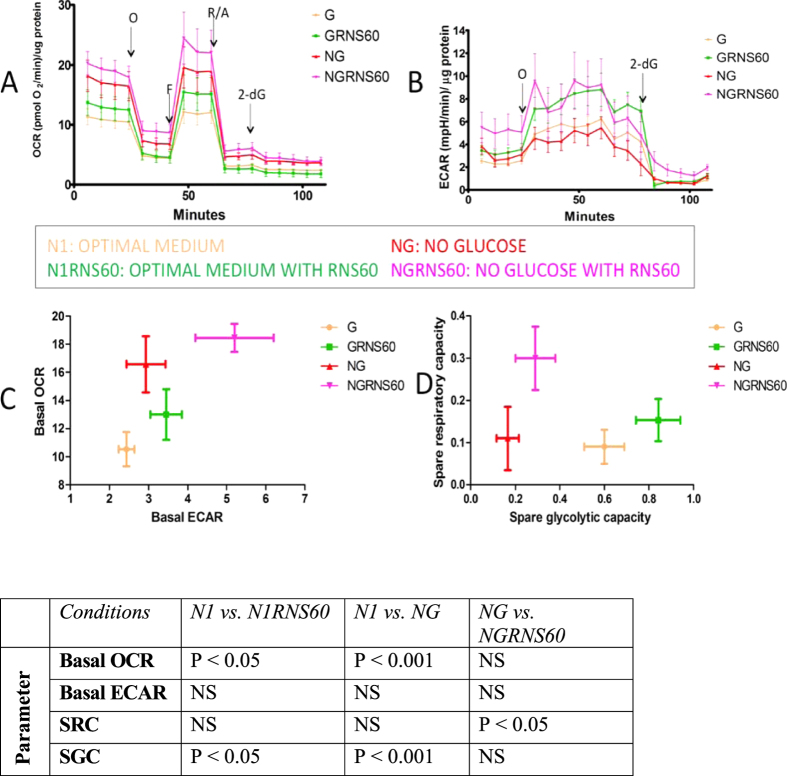
Influence of RNS60 on OCR and ECAR in of rat OLs cultured under optimal and glucose starvation conditions. (**A**) OCR plot of different treatments. (**B**) ECAR plot recorded concomitantly with OCR as in panel (a). (**C**) Basal OCR vs. basal ECAR [mean ± sem for both parameters]. (**D**) Spare Respiratory capacity vs. Spare Glycolytic capacity [mean ± sem]. PS – The statistical significance of comparisons depicted in (**C**,**D**) are tabulated. Abbreviations: O = Oligomycin, FCCP = carbonyl cyanide p-trifluoromethoxyphenylhydrazone, R/A = Rotenone/Antimycin A and 2-dG = 2-deoxyglucose (100 millimolar) NS = Not Significant.

**Figure 3:**
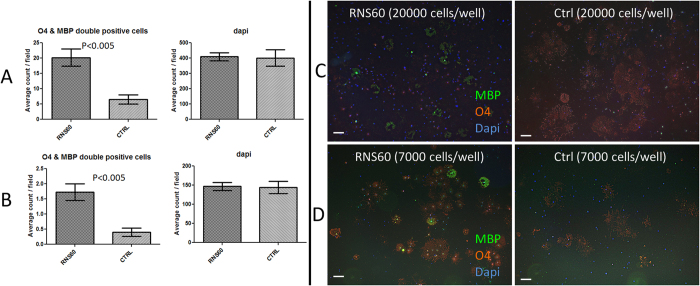
RNS60 promotes OPC differentiation. The average number of cells expressing the mature OL marker MBP was increased by RNS60 treatment. (**A**) Graphs illustrating the average O4-MBP double positive cells and average number of cells (dapi) per field for all the treatment conditions (plating density 20,000 cells/well). (**B**) Graphs depicting the average O4-MBP double positive cells and average number of cells (dapi) per field for all the treatment conditions (plating density 7,000 cells/well). (**C**) Micrographs depicting immunostaining for O4 and MBP (plating density 20,000 cells/well). (**D**) Micrographs depicting immunostaining for O4 and MBP (plating density 7,000 cells/well).[Scale bar ~100 μm].

Statistical analyses were performed in Graphpad Prism (v5.0). A one-way anova test was performed for comparisons shown in Fig. 1A, a two-way anova test was performed for comparisons in Fig. 2C,D and a t-test for comparisons in Fig. 3A,B. At least three biological replicates and five technical replicates were used. The statistical signifance, p values, are indicated in figures/table.

## Results

### RNS60 reduces caspase activation in rat OLs under metabolic stress

To determine whether RNS60 could protect OLs from metabolic stress induced injury, we assayed rat derived OLs deprived of glucose and nutrients. We used activation of caspase 3/7 as an index of cell injury. The percentage of the total number of cells that was caspase3/7- O4 double positive was significantly higher under glucose deprivation (NG) compared to control condition (G) (Fig. 1). Under the stress conditions applied here, OLs begin to activate caspases3/7 at 6 h with extensive activation by 35 h. At this time point we do not observe significant cell loss. In the NG condition, the addition of RNS60, significantly reduced the percentage caspase3/7- O4 double positive cells.

### RNS60 enhances bio-energetic properties of rat OLs in basal and stress conditions

To assess the influence of RNS60 on the bioenergetic properties of rat OLs under basal and metabolic stress conditions, we utilized a Seahorse bioanalyzer.

#### Effects under basal condition

In optimal cell culture conditions (XF conditions), the measured basal OCR was higher in the presence of RNS60 (XFRNS60) compared to media alone (Fig. 2A,C). No significant difference was detected in OCR between these two conditions after the addition of the mitochondrial inhibitors oligomycin, FCCP, Rotenone and Antimycin-A, indicating no significant change in oxygen consumption for mitochondria mediated ATP production (Fig. 2A). The SRC was assessed from the OCR achieved after the addition of FCCP. No significant difference in the SRC between the two conditions was detected. To assess OL glycolytic activity we measured ECAR simultaneously with OCR. Basal ECAR was increased after treatment with RNS60 (XFRNS60) compared to XF alone (Fig. 2B,C) indicating increased glycolysis. The SGC as determined by addition of oligomycin was also increased by RNS60 treatment (XFRNS60) compared to XF (Fig. 2D). As expected, addition of 2-DG resulted in a steep decrease in ECAR (Fig. 2B).

#### Comparison of basal and glucose-nutrient deprivation conditions

The basal OCR was increased in XFNG (glucose-nutrient deprivation) condition compared to XF (control) (Fig. 2A,C) an expected compensatory response by the cells. The OCR was higher in XFNG condition compared to XF after the addition of selective mitochondrial inhibitors oligomycin, FCCP, Rotenone and Antimycin A indicating higher non-mitochondrial respiration (eg. Glycolysis, fatty acid metabolism). The SRC did not differ between these two conditions further suggesting the use of non-glucose carbon sources (eg. Glutamine, pyruvate) by the cells under metabolic stress conditions. Basal ECAR was also not significantly different between the two. Consistent with expectations, XF exhibited a higher SGC compared to XFNG (Fig. 2D). The apparent steeper drop in ECAR in the XF respirogram compared to that of XF upon addition of 2-DG confirms higher glycolytic rate in the presence of glucose.

#### Effects of RNS60 under glucose-nutrient deprivation condition

Under our glucose-nutrient deprivation conditions (XFNG condition) basal OCR increased modestly after RNS60 treatment. Further, XFNGRNS60 had higher SRC compared to XFNG (Fig. 2D) indicating significant beneficial effects on mitochondrial metabolic properties. Basal ECAR was higher in RNS60 treated cells (XFNGRNS60) under stress compared to XFNG condition (Fig. 2B,C) reflecting glycolytic increment. The SGC did not significantly differ between the two conditions.

### RNS60 promotes OPC differentiation and maturation

**Figure 4:**
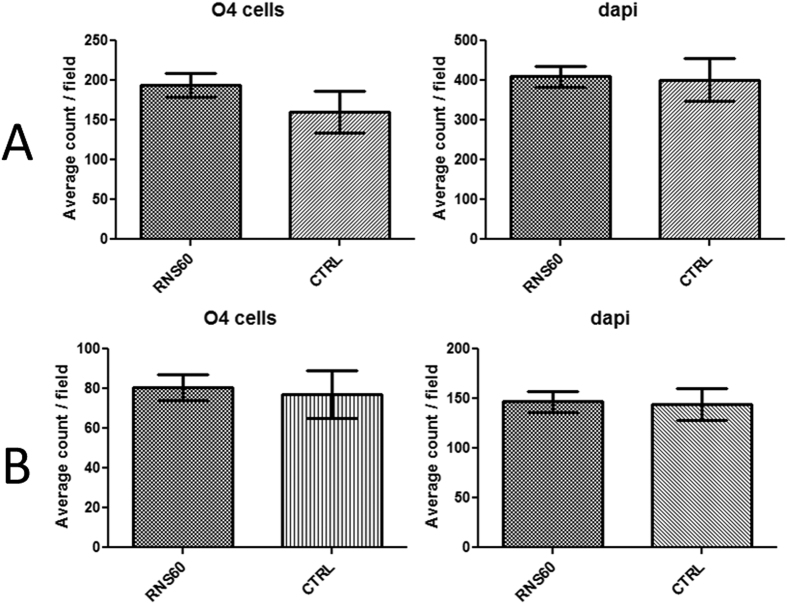
(**A**) Average counts of O4 expressing cells per field and average counts of dapi per field for the plating density of 20,000 cells per well on day 5 after plating. (**B**) Average counts of O4 expressing cells per field and average counts of dapi per field for the plating density of 7,000 cells per well on day 5 after plating.

To evaluate the effects of RNS60 on OPC differentiation we considered both early and late stages of differentiation under optimal conditions with and without RNS60. We utilized expression of O4 to identify early states of post-mitotic oligodendrocytes, and for late, more mature oligodendrocytes, used expression of MBP by O4 positive cells. After 5 days *in vitro* (DIV) the cultures in presence and absence of RNS60 contained ~50% O4+ cells [for both 20,000 and 7,000 cells/well plating densities] (Fig. 4). There was no siginificant increase in % O4 positive cells compared to cultures at 1DIV (data not shown). At 5 DIV, ~3% and <1% of the O4 positive cells were also immunopositive for MBP, in cultures plated at densities of 20,000 and 7,000 cells/wells respectively (Fig. 3A,B) under control condition. Interestingly, the average number of MBP expressing O4 positive cells per field was significantly higher when treated with RNS60 compared to the control condition at both plating densities. The average total number of cells per field did not significantly change between the two conditions (Fig. 3). At 1 DIV there were no MBP positive cells (data not shown).

## Discussion

The detrimental effect of metabolic stress on OPCs and OLs is an important feature of MS pathology. Hence, therapeutic interventions to reduce the effects of such stress may have beneficial effects. In search of such a therapeutic strategy, we tested RNS60, a potential modulator of respiration, for its prosurvival effects on OPCs and OLs *in vitro*. Under our metabolic stress conditions, RNS60 demonstrates beneficial effects in OLs. Moreover, under basal (physiological) conditions, the product promotes differentiation of OPCs.

### Protection from cell death

We chose glucose-nutrient starvation (NG) to model the metabolic stress conditions that would occur in the microenvironment of MS lesions. “Inside –out” lesions (also referred to as type 3 lesions) in MS cases feature cells undergoing apoptosis although the precise mechanisms of cell death remain to be defined. These changes resemble those found in focal ischemia^17^. In the ischemic brain, the extent of caspase activation determines the vulnerability of OLs to this metabolic insult^18^. Glucose deprivation stimulates intrinsic/mitochondrial apoptosis pathways, resulting in the activation of executioner caspases, caspases 3 and 7^19^,^20^. We subjected OLs to glucose/nutrient deprivation for 35 h to simulate ischemic metabolic stress associated with an MS plaque and assessed the activation of caspases 3/7. Previous reports have demonstrated that when OLs are deprived of oxygen, significant cell death can occur rapidly, within as little as 6 h^21^,^22^. In the specific conditions used here in our glucose-nutrient deprivation studies, significant cell loss had not yet occurred after 35 h and we selected this as a time point at which injury may be reversible.

### Respirometry reflects RNS60’s efficacy on OLs

Maintenance of myelin, a primary function of OLs necessitates these cells to have extremely high metabolic rates leading to consumption of large amounts of ATP^23^. Mature OLs account for the high myelin turnover rate in the human brain with little replacement by OPCs^24^. The energy requirements of OLs are at least 2 fold higher compared to other brain cells^25^,^26^. A plot of basal ECAR vs. OCR reveals an upward and rightward shift in the presence of RNS60 in optimal medium condition [XFRNS60 vs. XF] (Fig. 2C) indicating increases in both oxidative phosphorylation and glycolysis. The change in oxidative phoshorylation is in agreement with RNS60’s established ability to modulate the electrophysiological properties of the *Xenopus laevis* oocyte membrane by increasing mitochondrial-based ATP synthesis^27^. These findings suggest that RNS60 may enhance OL survival and function when challenged with conditions of metabolic stress.

Regarding the OL response to glucose-nutrient starvation, we observed an increase in basal OCR in XFNG condition compared to XF (Fig. 2C). This increase is comparable to observations in primary fibroblasts and cancer cells grown in medium in which glucose is replaced with galactose, showed an increased OCR compared to cells grown in medium containing a high concentration of glucose (25 mM)^28^,^29^,^30^. Basal ECAR did not significantly differ between XF and XFNG conditions. This can be attributed to potential ECAR contributions from metabolism of glutamine and pyruvate present in the XFNG medium. Basal ECAR significantly increased after RNS60 treatment, in the absence of glucose-nutrients (XFNGRNS60) (Fig. 2C). How RNS60 contributes to this ECAR increase needs further consideration. It can be speculated that RNS60 may improve the efficiency of metabolism of glucose-independent intermediates feeding into glycolysis. The basal OCR did not significantly differ between XFNG and XFNGRNS60 conditions (Fig. 2C).

Additionally, plotting spare glycolytic capacity (SGC) vs. spare respiratory capacity (SRC) revealed the effects of RNS60 conferring with basal OCR and ECAR measurements. Under induced metabolic stress condition (XFNGRNS60), RNS60 significantly enhanced oxidative phosphorylation and interestingly, under basal condition, it improved glycolysis. RNS60’s ability to impact mitochondrial ATP synthesis has also been shown in squid giant neurons^9^. The effect on glycolysis, as described, requires additional study.

### Promotion of OPC differentiation

Remyelination is frequently limited in recent MS lesions despite the presence of significant numbers of OPCs at the lesion edge. Various toxin induced demyelination models (cuprizone, lysolecithin etc) in rodents have shown that it is the OPCs rather than pre-existent OLs that underlie remyelination. OPCs are precursor cells that need to proliferate and eventually differentiate. “Increased mitochondrial function has been found to be beneficial for differentiation and maturation of OPCs because of the high energy consumption by developing OLs during myelination”^25^,^31^. We assayed OPC differentiation by quantifying the number of O4, GalC and MBP expressing cells under sub-optimal plating densities. RNS60 did not impact the number of cells expressing O4 and GalC assessed one day after plating (data not shown). However, 5 days after plating with two applications of RNS60, we detected higher numbers O4 and MBP expressing cells under RNS60 treatment compared to control medium (N1) condition. We believe our results are in line with many other studies that have attempted pharmacological promotion of OPCs differentiation^32^,^33^. Finally, RNS60 did not alter the rate of OPC proliferation.

## Conclusion

Addition of RNS60 *in vitro* promotes OLs survival and OPCs differentiation, supporting the possibility of potentially beneficial therapeutic applications for demyelinating conditions.

## Additional Information

**How to cite this article**: Rao, V. T. S. *et al*. Potential Benefit of the Charge-Stabilized Nanostructure Saline RNS60 for Myelin Maintenance and Repair. *Sci. Rep*. **6**, 30020; doi: 10.1038/srep30020 (2016).
